# Human-in-the-loop AI predictive digital twin to extend virtual precision diabetes care between visits

**DOI:** 10.1038/s44401-026-00118-8

**Published:** 2026-07-08

**Authors:** Jing Wang, Syed Hasib Akhter Faruqui, Adel Alaeddini, Yan Du, Shiyu Li, Yijiong Yang, Kumar Sharma

**Affiliations:** 1 College of Nursing, Florida State University, Tallahassee, FL USA; 2 Department of Engineering Technology, Sam Houston State University, Huntsville, TX USA; 3 Department of Mechanical Engineering, Southern Methodist University, Dallas, TX USA; 4 The University of Texas Health San Antonio, San Antonio, TX USA; 5 School of Kinesiology, Louisiana State University, Baton Rouge, LA USA

**Keywords:** Computational biology and bioinformatics, Diseases, Endocrinology, Health care, Medical research

## Abstract

In a 6-month randomized trial, we evaluated a digitally enabled “human-in-the-loop” care support model using a predictive artificial intelligence (AI) digital twin to provide personalized daily short message service (SMS) feedback for adults with type 2 diabetes (T2D). The parent study enrolled 40 adults aged ≥18 years with T2D who completed 3 months of baseline observation followed by a 3-month intervention period, generating 6467 longitudinal data points across weight, dietary intake, physical activity, and glucose monitoring (mean follow-up: 174 days). For this ancillary AI intervention, a subset of 19 participants was randomized to receive either AI-generated individualized daily feedback (AI group, *n* = 10) or no daily feedback (control group, *n* = 9). The online human-in-the-loop predictive control model incorporated a transfer-learning artificial neural network predictive digital twin trained on participant self-monitoring data, including weight, food logs, physical activity, and glucose values. A particle swarm optimization controller identified personalized behavioral recommendations aligned with glucose and weight goals, and the digital twin was retrained weekly using newly accrued data. The model achieved ≥80% prediction accuracy across all diet-condition subgroups. During the intervention period, participants receiving AI-generated feedback demonstrated trends toward increased daily step counts and improved adherence to caloric and carbohydrate intake targets. The AI intervention group achieved significantly greater weight loss than controls (mean loss 5.87 lbs vs 3.57 lbs; *p* < 0.012) while maintaining stable glucose levels throughout the study period (*p* = 0.661). These findings suggest that AI-enabled predictive digital twin models may offer a scalable approach for extending precision diabetes self-management support beyond clinic visits.

## Introduction

Type 2 diabetes (T2D) is a leading chronic condition worldwide and a major driver of preventable complications, healthcare utilization, and costs^1^. Effective management requires sustained lifestyle support and frequent self-management decisions across diet, physical activity, and medication adherence^2^. However, many health systems face challenges in delivering continuous, individualized coaching between clinic visits because of workforce shortages, limited visit capacity, and unequal access to diabetes education - factors that may exacerbate outcome disparities^3,4^.

The digital health interventions can extend self-management support beyond the clinic, but many solutions deliver generic recommendations that are insufficiently tailored to individual patterns and preferences^5^. Precision approaches that learn from a person’s longitudinal self-monitoring data may improve relevance and engagement while reducing clinician burden. “Digital twin” models, computational representations that predict near-term physiologic outcomes based on behavior inputs, offer a pathway to individualized lifestyle guidance^6–8^. Transfer learning can further enable such models to be trained in data-sparse settings by leveraging shared patterns across patients and then fine-tuning to subgroup- or individual-level characteristics^9^.

Most algorithmic approaches in diabetes management have focused on predicting glucose trajectories or automating insulin delivery^10–12^, with relatively limited studies integrating AI-based prediction designed to support lifestyle behavior change in real-world care delivery contexts^13,14^. For health systems, the key question is not only whether an algorithm predicts outcomes accurately, but whether it can be operationalized as a low-burden intervention that improves clinically meaningful outcomes, maintains safety (e.g., glycemic stability), and scales across populations^15^.

In this study, we implemented a predictive digital twin (PDT) framework, which represents a data-driven approximation of a patient’s short-term physiological response to lifestyle behaviors. Unlike fully realized digital twins that incorporate bidirectional coupling, mechanistic modeling, and real-time synchronization with physical systems, the PDT used here focuses on predicting near-term outcomes (e.g., next-day glucose and weight) based on observed inputs. This approach retains key elements of the digital twin paradigm, including individualized modeling and iterative updating using longitudinal data, while remaining computationally feasible for real-world clinical deployment. We evaluated a human-in-the-loop AI intervention that uses a transfer-learned predictive digital twin and an online optimization controller to generate daily, personalized text-messaging feedback to adults with T2D. We conducted a randomized trial to assess early effectiveness on lifestyle behavior trends and weight outcomes while monitoring glycemic stability, and we describe the intervention as a health system–relevant approach for extending precision diabetes self-management support between visits.

## Methods

### Study design and setting

This study was a randomized trial embedded within a single-center parent randomized controlled trial (RCT)^16^. The parent trial enrolled overweight/obese adults and followed participants longitudinally using mobile health devices and daily self-monitoring. The present trial evaluated an ancillary AI-enabled intervention delivered during the second half of the study period^16^.

### Participants and data collection

The parent trial enrolled 60 overweight or obese adults (≥18 years), including 40 participants with T2D according to the American Diabetes Association criteria^16^. Participants were assigned to a ketogenic or low-fat, low-calorie diet and self-monitored dietary intake using a smartphone-based application. Physical activity was tracked using a *Fitbit Inspire 2*, and body weight was measured using a *Withing’s* body scale. All participants received health education consistent with evidence-based behavioral lifestyle interventions. Participants completed a 3-month baseline observation period followed by a 3-month intervention (mean follow-up: 174 days), generating 6467 longitudinal daily data points from self-monitoring of dietary intake, body weight, physical activity (steps), and glucose-related measures^17^. These data were used to support transfer learning–based pretraining of a predictive digital twin model.

For the ancillary AI intervention, participants were selected from the subset of individuals with type 2 diabetes who had complete longitudinal self-monitoring data across both the baseline and intervention phases. A total of 19 participants met these criteria and were included in the present analysis. These participants were first stratified based on their study group assignment in the parent trial and relevant health conditions (e.g., with or without early-stage chronic kidney disease), and then independently randomized into the AI intervention group (*n* = 10) or control group (*n* = 9) using a simple random allocation procedure. This randomization was conducted separately from the parent trial assignment, ensuring that the AI intervention represented an independent experimental comparison. Data from the initial 3 months were used to train the predictive digital twin, after which continuously streamed data informed an online human-in-loop predictive control (OHLC) model that generated daily recommendations delivered via short message service (SMS). The predictive model was retrained weekly to improve performance. Baseline demographic and clinical characteristics were assessed to ensure comparability between the AI and control groups.

### Model construction: Predictive digital twin (transfer-learned ANN)

The PDT at its core utilizes an artificial neural network (ANN) predictive digital twin model that has been trained using transfer learning (TF) strategies to improve its performance (for both data-rich and data-scarce setups) to generate food intake suggestions^18,19^. As proof of concept, we have the following contributions in this work:

- A human-in-the-loop artificial intelligence feedback system.
- A transfer-learned predictive digital twin model of patients.
- An evolutionary optimization-based online control model in mHealth devices.

Human-in-the-loop oversight was incorporated as a safety and clinical validation layer within the intervention. All AI-generated recommendations were reviewed by a trained nurse or interventionist prior to delivery to participants. The reviewer evaluated the clinical appropriateness, feasibility, and safety of suggested dietary and activity targets, including specific food items and portion sizes. Based on this assessment, a penalty score ranging from 1 (highly appropriate) to 1000 (clinically inappropriate) was assigned and incorporated into the optimization objective function. This process ensured that recommendations aligned with established clinical guidelines and patient-specific considerations before dissemination via SMS.

**Fig. 1:**
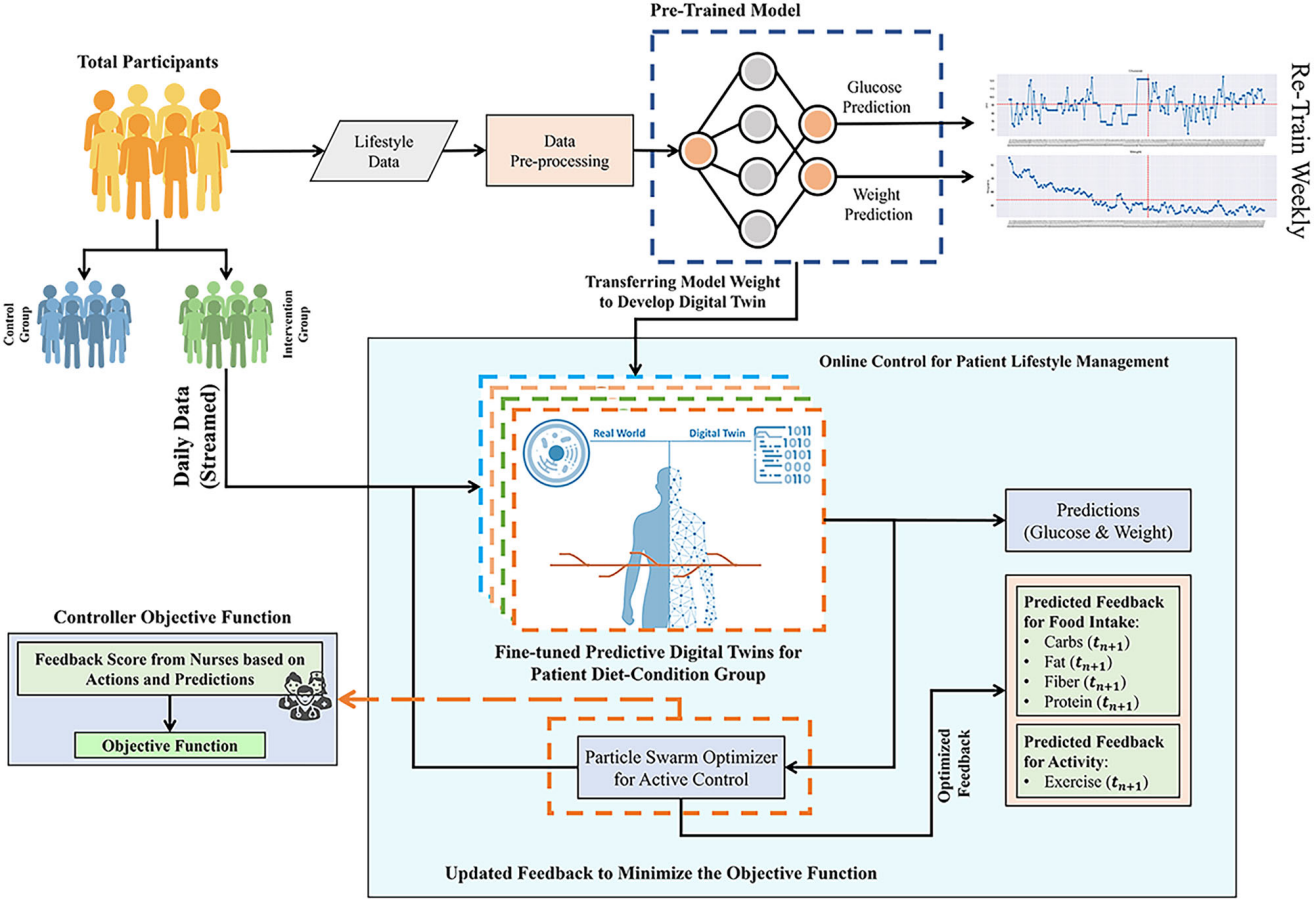
Overview of the proposed digital twin framework for personalized lifestyle intervention. Lifestyle data collected from participants were preprocessed and used to train a deep learning model for glucose and weight prediction. Transfer learning was applied to develop personalized digital twins, which generated optimized dietary and activity feedback through an online control system for individualized lifestyle management.

Figure 1 shows the overall schema of the proposed model. We first build a predictive model where inputs are the daily lifestyle choices, and the outputs are the next day’s glucose and weight levels. Next, we used the predictive model as the core block for the online control model. The control model is built based on the Particle Swarm Optimization (PSO) method, which generates feedback that minimizes glucose and weight outputs. We retrained the models every week. PSO utilizes the fine-tuned model as a core plant to generate dietary suggestions. We employed a pretrain-then-fine-tune transfer learning strategy, in which the model was first trained on pooled data from all participants to learn shared physiological patterns, and subsequently fine-tuned on subgroup-specific data defined by diet and clinical condition.

### Predictive digital twin: transfer learning-based artificial neural network to predict patient states

The intervention was designed as a “between-visit” self-management support model. During the 3-month baseline phase, no participants received AI feedback. At the start of the 3-month AI-intervention phase, the AI group began receiving daily personalized SMS containing actionable lifestyle suggestions (e.g., dietary and activity adjustments) generated by the OHLC model. The control group continued usual self-monitoring and diet guidance without daily AI SMS.

The SMS-based intervention consisted of three integrated components. First, personalized dietary recommendations were generated using the predictive digital twin and PSO optimization framework, which produced individualized macronutrient targets that were subsequently translated into specific meal plans using a linear programming approach. Second, the domain of behavioral messaging (e.g., dietary adherence, physical activity encouragement) was informed by the AI-generated targets. Third, motivational message content was selected from a predefined library by the nurse reviewer based on clinical judgment and patient context.

Participants received one SMS message per day containing these combined elements. The system was unidirectional, and participants did not interact directly with the AI system. Adherence to recommendations was not formally quantified but was indirectly assessed through longitudinal behavioral trends.

We start by developing a PDT that will predict next-day blood glucose and body weight based on observed dietary intake, physical activity, and prior physiological state. By definition, a predictive twin models the future behavior and state of any system in consideration. These models utilize historical data to incorporate possible system modes within the model. We construct the PDT to receive self-monitoring data from patients (food intake, weight, exercise regimen, etc.). This allows the twin to simulate the physical object in real-time, in the process offering insights into their performance (weight and blood glucose control).

We construct a simple multilayer perceptron/artificial neural network (with three hidden layers) (ANN) for predictive modeling of daily glucose levels using the health time series data. We utilize a transfer learning strategy to take care of the missing values. In literature, transfer learning strategies have proved to make learning from data efficient, fast, and under sparse data fruitful^19^. To achieve this goal, we concatenated and sampled all the patient’s data and created the transfer learning dataset to pre-train the ANN model^20^. Once the pre-training is done, the model is fine-tuned for each patient diet-condition group (*1. Keto Diet – Obese and T2D, 2. Keto Diet – Obese, Kidney Disease, and T2D, 3. Low-Fat Diet – Obese and T2D, 4. Low-Fat Diet – Obese, Kidney Disease, and T2D*). The training procedure involves sampling data to train a deep learning model for each patient group of interest, where the ANN model weights of the pre-trained model are used as the prior. We compare the performance of the model with classic machine learning models (*KNN, Gaussian Process, Decision Tree, Random Forest, Gradient Boosting*). Parameters for all the models are tuned using a grid search approach. While many of the above-mentioned algorithms can be used for building the predictive twin (comparable accuracy; later shown in the results section), we selected the transfer learning (TF)-based ANN algorithm for our analysis for two specific reasons:

- They adapt to learning complex patterns present in the data, and
- The predictive twin can be trained even if there is a low amount of data (data sparsity) for a new patient group.

**Fig. 2:**
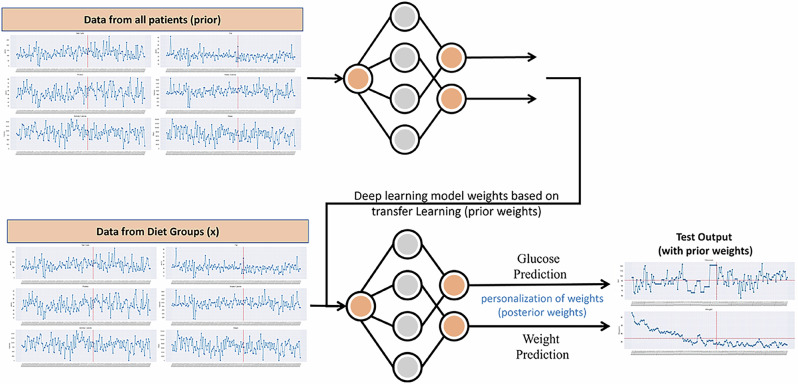
Scheme of the transfer learning strategy to train an artificial neural network for predictive modeling. The upper panel illustrates pretraining of the deep learning model using data from all patients to establish prior model weights. The lower panel demonstrates transfer learning and personalization of the model using data from diet-specific groups to generate posterior weights for individualized prediction. The trained model was used to predict glucose and weight outcomes.

Figure 2 shows a visual representation of the transfer learning strategy used in this work. We followed the same steps as Faruqui et al.^14^ The only change was that they fine-tuned their models for each patient, and we fine-tuned for each patient’s diet-condition group.

The input features of the predictive model included daily dietary macronutrient intake (carbohydrates, fat, fiber, and protein), physical activity (daily step count), and prior-day physiological measurements (body weight and blood glucose levels). Temporal dependencies were incorporated through the inclusion of lagged variables rather than recurrent neural network architectures.

The artificial neural network was trained using the Adam optimizer with a learning rate of 1 × 10⁻⁵ for 200 epochs. No dropout or explicit regularization techniques were applied. Model training and evaluation followed a sequential design, with the first three months of baseline data used exclusively for training and a subsequent non-overlapping period used for testing, thereby avoiding data leakage.

### Online control model: particle swarm algorithm

Once the PDT is developed, we focus on developing the control model of the proposed algorithm. The control model uses PDT (of the patient-diet group) to determine what combination of food intake and exercise would help a patient maintain their blood glucose level and help them lose weight while maintaining their blood glucose level. This highly non-linear problem needs to be optimized efficiently within the search space. We deploy the Particle Swarm Optimization (PSO)^21^ algorithm as the controller due to its simple implementation; it doesn’t need a gradient form of the objective function and, depending on the setting, is less memory consuming (can be used in both online and off-line setup). For a function defined in a multidimensional vector space (as in our study case), PSO is a robust meta-heuristic optimization algorithm and one of the best optimization algorithms to find the minimum or maximum of the objective function. The PSO algorithm (Step 2 of Fig. 1) is then used with the predictive twin to determine the best parameters to minimize the objective function. The PSO algorithm consists of two components: (1) the Objective function and (2) the constraints.

The OHLC controller used particle swarm optimization (PSO) to identify feasible behavior changes that minimize predicted risk while supporting weight goals and glycemic stability. Constraints for macronutrient recommendations were defined based on diet requirements and clinical team input, enabling clinically sensible search boundaries. Human-in-the-loop input was incorporated as a penalty factor that increased the cost of implausible or undesirable recommendations, allowing nursing expertise to improve safety and appropriateness. The PDT was retrained weekly during the intervention period to incorporate newly accumulated data and adapt guidance over time.

### The objective function

To set up PSO for our study, we need an objective function that helps us achieve our goal. For a patient in the low-fat diet group, our goal is to suggest a food and exercise regimen that can help them lose weight (*W*) till they reach their target weight level $\left({W}_{G}={W}_{0}-0.2{W}_{0}\right)$. Once they reach their target level, we switch the goal to maintaining that weight. The patients will also need to maintain their Glucose levels (*G*) within an acceptable range $\left(70\le G\le 130\right)$. A patient in the keto diet group must attain (or close to) a certain blood ketosis level. To do that, they need to reach a keto ratio (*K*) of 1.5 or higher. The keto ratio can be computed from the suggested food intake (macronutrients) by the PSO algorithm. The objective function is special in the sense that we don’t want the algorithm to select extreme parameters (in our case, food intake and physical activity suggestions) that cause the glucose level to go near zero or too high a ketone level. To resolve this issue, we introduced a tuning parameter *λ*, which takes the value of 0 whenever a generated suggestion reaches its desired value; otherwise, it takes a value of 1. This helped control the model to achieve its desired output. We integrated the feedback of the nursing knowledge using a second multiplier (penalty factor, *m*) for each element of the objective function. Depending on the suggestion provided by the controller, if the predicted glucose/weight/ketone level is too diverse, a nurse can assign a higher penalty and vice versa. In our case, this assigned score ranged from 1 to 1000 to penalize the model if it’s too far away from desired values. At the end of each evaluation phase, the score derived from nursing knowledge is assigned to the model. The details of the decision boundaries considered by the nurses are provided in Appendix 1. Thus, considering the above, the objective function for the PSO algorithm is set as follows:

Objective Function

$$\mathop{\min }\limits_{{\rm{l}}=1,\,\ldots .,{\rm{L}}}\mathop{\sum }\limits_{i=1}^{n}{\lambda }_{1}{{m}_{1}\hat{G}}_{i}+{\lambda }_{2}{m}_{2}{\hat{W}}_{i}+{{\rm{\lambda }}}_{3}{m}_{3}\left(1.5-{{\rm{K}}}_{{\rm{i}}}\right)$$

where,

Tuning parameter,

- $${\lambda }_{1}=0$$; if $$70\le {\hat{G}}_{i}\le 130$$
- $${\lambda }_{2}=0$$; if $$\left({W}_{i}-\hat{{W}_{i}}\right)\ge 0$$ or $${W}_{G}=\hat{{W}_{i}}$$
- ${\lambda }_{3}=0$; if Patient-Diet Group=‘keto-diet’ or $\left(1.5-{K}_{i}\right)\ge 0$
- ${m}_{i=\{1,\,2,\,3\}}$=Score assigned by a human-in-the-loop. This is used in conjunction with the tuning parameters $\left({\lambda }_{i=\{\mathrm{1,2,3}\}}\right)$
- $K=\frac{{f}_{a}}{(c-{f}_{b}+p)}$; *K* = Keto ratio, *c*= carb, *f*_*a*_ = fat, *f*_*b*_= fiber, *p*= protein
- ${W}_{G}={W}_{0}-0.2{W}_{0}$; *W*_*G*_*=* Weight Goal, *W*_0_= Patients weight at the start of the study

The scores (*m*) assigned by the human in the loop can also be automated in future studies by:

1. Building a lookup table generated by the nurse/s for all the factors to be used during the optimization session.
2. Building an approximating linear function by using the lookup table. That way, we can approximate the penalty factors even if there is a value present that was not assigned a penalty by the nurse/s.

### The constraints

Furthermore, we also define the search space for the proposed model. The model constraints are decided based on the appropriate diet requirement^22,23^ and discussions with humans (nurse-led research team) in the loop. The constraints may also be adapted (*exception case*) based on patient-specific needs, based on their physical condition and nutritional needs, thus making the model adaptive not only patient group specific but also patient specific. For this work, the general constraints were defined as follows in Table 1:

**Table 1:** General constraints for low-fat and ketogenic diet groups

Low-fat group	Keto group
$195\le c\le 300$	$20\le c\le 50$
$20\le {f}_{a}\le 55$	$90\le {f}_{a}\le 200$
$20\le {f}_{i}\le 50$	$20\le {f}_{i}\le 50$
$100\le p\le 160$	$30\le p\le 110$

### Outcomes

The primary study outcomes were (1) weight change during the intervention period and (2) glycemic stability over time. Model performance was evaluated using Clark Error Grid analysis, reported as the percentage of predictions falling in Zone A. Glycemic stability was operationalized as the absence of statistically significant changes in mean glucose levels within each group from baseline to end of study, as assessed using repeated measures analysis.

### Statistical analysis

We used ANOVA to evaluate intervention effects on weight change over time and to assess glucose stability across the study period. Model performance comparisons were summarized using Clark Error Grid Zone A accuracy across diet-condition subgroups. Due to the small sample size, statistical analyses were considered exploratory. Model assumptions for ANOVA were assessed, and results were interpreted with caution, given limited statistical power. Reported *p*-values reflect both within-group changes over time and between-group differences, as specified in each result.

### Ethics and dissemination

This study was conducted in accordance with the ethical principles of the Declaration of Helsinki and in compliance with U.S. federal regulations for the protection of human subjects (45 CFR 46). The study protocol was reviewed and approved by the Institutional Review Board of the University of Texas Health Science Center at San Antonio (Protocol Number: HSC20190528H). Written informed consent was obtained from all participants (in related files). The study was registered at http://ClinicialTrials.gov (NCT05071287). The initial release date was 2021-06-03.

## Results

### Demographics

The sample included 19 participants, of whom 52.6% identified as male and 47.4% as female. Most participants identified as Hispanic White (47.4%). Educational attainment varied, with 42.1% reporting some college, 26.3% holding a graduate degree or higher, and 31.6% having a high school diploma or college degree. Nearly half of the participants reported a household income of $60,000 or more (47.4%). The majority were married (68.4%), while 21.1% were divorced and 10.5% never married (**See** Table 2).

**Table 2:** Demographic and socioeconomic characteristics of participants

Socio-demographics	Total (*N* = 19)
**Age, Mean (SD)**	58 (10)
**Gender Identity,** *N* **(%)**	
Male	10 (52.6%)
Female	9 (47.4%)
**Race,** *N* **(%)**	
Hispanic White	9 (47.4%)
Non-Hispanic White	6 (31.6%)
Hispanic Native American	2 (10.5%)
Non-Hispanic Native American	0 (0)
Hispanic other	2 (10.5%)
**Education Level,** *N* **(%)**	
High school graduate	3 (15.8%)
Some college	8 (42.1%)
College graduate	3 (15.8%)
Graduate degree or higher	5 (26.3%)
**Household Income,** *N* **(%)**	
Under $20,000	3 (15.8%)
$20,000–$39,999	3 (15.8%)
$40,000–$59,999	4 (21.1%)
$60,000 or more	9 (47.4%)
**Marital status,** *N* **(%)**	
Never married	2 (10.5%)
Married	13 (68.4%)
Divorced	4 (21.1%)

### Evaluation of predictive digital twin performance

We considered two variations of the proposed PDT model. The two variations are (1) The base ANN (without transfer learning) and (2) ANN-TF (with transfer learning). Several baseline methods are used to compare the performance of the proposed model. All the models are tuned using a grid search approach.

To evaluate the performance of the proposed model, we trained it using the first three months of data, during which no intervention was performed in both groups. We used one month of test data immediately following the third month’s intervention. Although not explicitly included in the reported evaluation (as shown in Table 3), the PDT utilized for the control model was re-trained at the end of each week as the patient’s new data became available. We evaluated all the models using Clark Error Grid^24^. Table 3 summarizes the percentage of prediction that falls on Zone A of the Clark Error Grid for all comparing methods.

**Table 3:** Prediction accuracy of the proposed neural network models along with other comparison methods based on Clark Error Grids zone A

Model	Keto Diet Group		Low-Fat Diet Group	
	Obese + Diabetes	Obese + Kidney + Diabetes	Obese + Diabetes	Obese + Kidney + Diabetes
	Glucose Level	Glucose Level	Glucose Level	Glucose Level
**KNN Regression (Weight: Distance)**	77.50%	77.50%	77.50%	77.50%
**KNN Regression (Weight: Uniform)**	77.78%	77.78%	77.78%	77.78%
**Gaussian Process (Kernel: RBF)**	76.94%	76.94%	76.94%	76.94%
**Decision Tree**	71.67%	71.67%	71.67%	71.67%
**Random Forest**	84.17%	84.17%	84.17%	84.17%
**Gradient Boosting**	84.44%	84.44%	84.44%	84.44%
**Artificial Neural Network (ANN)**	79.72%	87.44%	87.78%	84.23%
**ANN (with Transfer Learning)**	80.00%	87.85%	87.22%	84.90%

The proposed model, ANN-TF, performs the best, followed by ANN, Gradient Boosting, and Random Forest. Both Random Forest and Gradient Descent showed significant performance (84.17% and 84.44%) in predicting the blood glucose level for patients in the Keto-Diet group with obesity and Diabetes. They are competitive with the proposed ANN-TF model for patients with a Low-Fat Diet group with obesity. Kidney disease and Diabetes (84.17% and 84.44%). Compared to these, the all-other method falls below 80% in performance, especially the Decision Tree, which has the lowest prediction performance (71.67%) across all the patient groups. Therefore, based on the evaluation in Table 3 (also considering the advantage of transfer learning whenever the data is sparse) and ease of use for control steps, we decided to utilize the ANN-TF model as the PDT for the control model.

### Evaluation of the control model

To evaluate the performance of the control model and its effect on patients, we separated the study patients into two sub-groups (as mentioned earlier).

- Group 1 started receiving feedback from the proposed model daily (intervention group), and
- Group 2 did not receive any feedback from the model (control model). The participants didn’t receive any feedback for the first three months of the study.

After the first in-person intervention (at month three), group 1 started receiving daily feedback SMS. Group 2 continued to follow their suggested diet (the same diet they followed for the first three months).

**Fig. 3:**
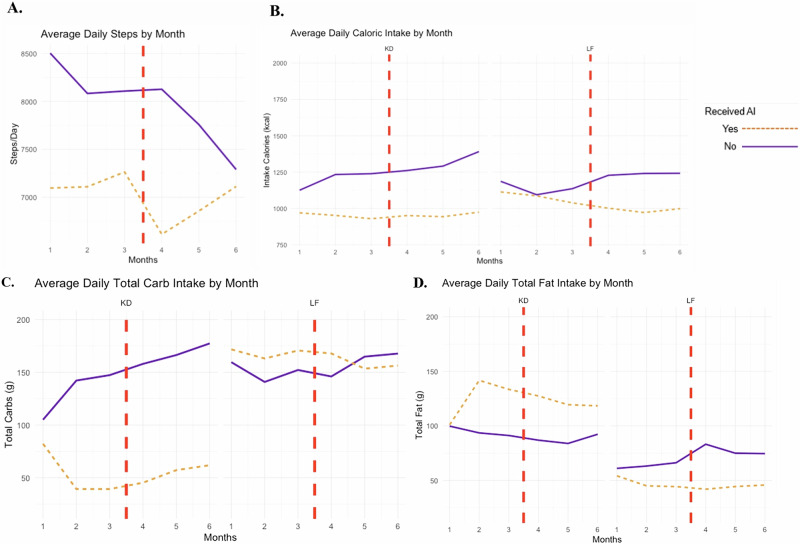
Self-monitored lifestyle behaviors over time in the keto (KD) and low-fat (LF) groups. Self-monitored lifestyles data of **A** average daily steps by month; **B** daily caloric intake by month between the keto (KD) and low-fat (LF) group; **C** daily total carb intake by month between the KD and LF group; and **D** daily total fat intake by month between the KD and LF group. Red dash vertical lines meant the time point (3-month) AI SMS started to be sent out.

This was an online setup, so we continued to receive patients' self-monitoring data daily, and based on that, we continued to provide them with daily food and exercise feedback for three months. Fig. 3 shows the evaluation results of self-monitored lifestyle data for the patients in this study between the two groups. It shows the average monthly breakdown of the collected self-monitored data, including patients’ average daily steps, daily caloric intake, total carb, and fat consumed across two distinct groups among the patients adhering to either a ketogenic diet or a low-fat diet.

### Clinical outcomes: weight change and glycemic stability

**Fig. 4:**
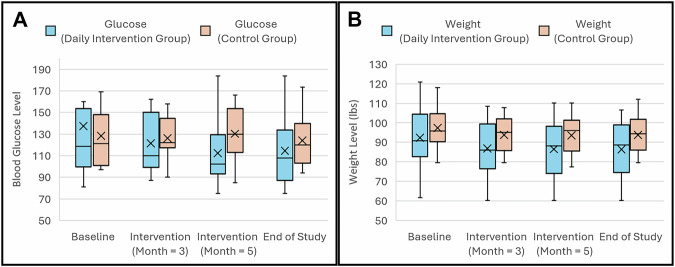
Changes in blood glucose and body weight between the AI intervention and control groups. Weight and glycemic change of AI and non -AI Groups. **A** Blood glucose levels at baseline, Month 3, Month 5, and end of study in the daily intervention (AI) group and control group. **B** Body weight changes over the same study period in both groups. Box plots represent median, interquartile range, and minimum/maximum values; × indicates the mean.

The non-AI (control) group demonstrated a modest but statistically significant reduction in body weight (mean decrease = 3.57 lbs., *p* = 0.013), while the AI intervention group experienced a larger and statistically significant weight reduction (mean decrease = 5.87 lbs., *p* < 0.012). Although weight loss occurred in both groups, the magnitude of reduction was greater in the AI intervention group. In contrast, glucose levels remained stable over time in both the AI and non-AI groups, with no statistically significant changes observed from baseline (*p* > 0.05). With respect to goal attainment, four participants in the AI group achieved their assigned weight goals, including the highest observed weight loss of 14.82%, compared with two participants in the non-AI group, whose maximum weight loss was 8.36%. Overall, these findings suggest that AI-generated feedback may enhance engagement and support greater adherence to lifestyle modifications, particularly with respect to weight management, while maintaining stable glucose levels (See Fig. 4).

## Discussion

The objective of this study was to evaluate the effectiveness of an OHLC integrated with a predictive digital twin (PDT) in delivering continuous, individualized feedback to adults with T2D to support adherence to healthy lifestyle behaviors between clinic visits. To achieve this aim, we developed a transfer learning–enabled artificial neural network (ANN-TF) that serves as the core of the PDT. Leveraging patients’ self-monitored data, the PDT learns individualized patterns of glucose and weight dynamics, enabling the development of adaptive control models that more accurately estimate behavioral and physiological targets relevant to weight and glycemic management.

By integrating the PDT with a particle swarm optimization–based control algorithm, the OHLC generates dynamic, personalized recommendations tailored to each patient’s lifestyle, dietary patterns, and physical activity levels. This personalization is critical for identifying effective interventions in T2D management, where inter-individual variability substantially influences outcomes^25^. Nurse-assigned scores were incorporated into the loss function as a human-in-the-loop safeguard to align AI-generated recommendations with clinical judgment and support steady, sustainable behavior change. Among evaluated modeling approaches, ANN-TF demonstrated the strongest predictive performance, followed by the base ANN, gradient boosting, and random forest models, all of which may serve as viable PDT architectures depending on computational constraints.

Analysis of self-monitored data revealed distinct differences between AI and non-AI groups in physical activity, dietary intake, and weight outcomes. Participants receiving AI-generated feedback achieved significantly greater weight loss than controls, indicating the effectiveness of the OHLC framework in promoting behaviors conducive to weight management. Importantly, glucose levels remained stable across both groups, suggesting that improvements in weight and lifestyle adherence were achieved without compromising glycemic control.

This randomized trial evaluated a human-in-the-loop, AI-enabled digital twin intervention delivered via daily SMS, designed to extend precision diabetes self-management support between clinic encounters. In the context of health systems facing workforce shortages and limited visit capacity, the intervention represents a scalable care-delivery approach that may augment traditional diabetes management without requiring continuous synchronous clinician involvement. The robust performance of the transfer-learned PDT across diet-condition subgroups further supports its applicability in settings with limited individual-level data.

While the study was not designed to formally evaluate behavioral mediation pathways, observed trends in physical activity and dietary intake suggest that AI-generated recommendations may influence behavioral adherence, which in turn contributes to improved weight outcomes. These findings provide preliminary support for the hypothesized pathway linking prediction, optimization, behavioral modification, and clinical outcomes.

From a health systems perspective, several features enhance potential implementation. SMS-based delivery minimizes access barriers and integrates readily into existing chronic disease management workflows; weekly model retraining enables ongoing personalization as patient data evolves; and human-in-the-loop penalties provide a safety layer that complements clinical oversight. The system is intended as a supportive coaching extension rather than a replacement for clinician decision-making.

Future research should evaluate implementation and equity outcomes critical to health system impact, including patient engagement and adherence to self-monitoring, differential effectiveness across subgroups, workflow burden associated with oversight and escalation, and downstream utilization outcomes such as acute care visits, medication changes, and total cost of care. Multi-site studies with longer follow-up are needed to assess sustainability, scalability, and effects on quality metrics relevant to payers and healthcare delivery systems.

This study has limitations. First, the trial included a small sample (*n* = 19) from a single center, limiting statistical power, generalizability, and increasing the risk of imbalance between groups^26^. Accordingly, findings should be interpreted as exploratory and proof-of-concept rather than definitive evidence of clinical effectiveness. Second, the study design and small sample size do not allow us to distinguish the specific effects of AI-driven personalization from the general impact of receiving daily behavioral prompts, and behavioral change mechanisms were not formally assessed using validated instruments or mediation analyses. Third, the follow-up period was relatively short, and the long-term sustainability of behavior change remains unknown. Finally, glucose and weight outcomes are influenced by factors beyond lifestyle behaviors, including medications, comorbidities, and social determinants of health, which were not fully modeled in this study. Together, these limitations suggest that results should be interpreted as early evidence supporting the feasibility and potential effectiveness of a scalable between-visit support model.

This study suggests that a human-in-the-loop AI predictive digital twin intervention delivered via daily SMS may support weight management while maintaining glycemic stability in adults with T2D, offering a potentially scalable approach for extending precision diabetes self-management support beyond clinic visits. By integrating transfer learning, online optimization, and nursing-informed safeguards, the model represents a potentially deployable digital health strategy for health systems seeking low-burden interventions to strengthen chronic disease management. Larger multi-site studies are needed to establish intervention effectiveness and evaluate clinical, implementation, equity, utilization, cost, and quality outcomes to determine real-world health system value.

## Supplementary information

Appendix

## Data Availability

The data collected for this study, including individual participant data, will be made available upon request. The data will be shared in a deidentified format to ensure participant privacy and confidentiality. Access to the data will be granted following the approval of a detailed proposal and the signing of a data access agreement. This agreement will outline the terms of use, ensuring that the data are utilized for legitimate research purposes and in a manner that preserves the confidentiality and integrity of the data. Requests for data should be directed to Yan Du. The study protocol, statistical analysis plan, and informed consent form are accessible in the published protocol paper associated with this study.

## Code availability

The code underlying the predictive digital twin and online human-in-the-loop control algorithm is not publicly available due to institutional and data security restrictions, but may be made available to qualified researchers upon request to the corresponding author.
