# Supplementary material for: Human-in-the-loop AI predictive digital twin to extend virtual precision diabetes care between visits
Source: Npj Health Syst. 2026 Jul 8;3:59. doi: 10.1038/s44401-026-00118-8 (PMC13354173; doi:10.1038/s44401-026-00118-8)
Supplement: Supplementary file 1 — Appendix [file 44401_2026_118_MOESM1_ESM.docx]

# **Appendix 1:**

## **1.1. Model Scoring**

The AI prediction ability was determined by the interventionists using a rating scale of

1. Bad (score: 1000),
2. Okay (score: 500),
3. Good (score: 100), and
4. Very Good (score: 1).

The important levels of A.I. predicted variables (e.g., intake calories, fat in grams, weight) were classified into 3 categories.

1. Very Important,
2. Moderately Important, and
3. Low Importance.

Figure A.1. below illustrates the workflow the nurse-in-the-loop used to assess and enhance the A.I. model's performance over time for the subjects in the study. The boundaries set for each subject is based on the in-person assessment done when the study started (the actual boundaries not shown in the appendix). The hard boundaries set for each ­­variable had to be met depending on participant’s diet group and personal characteristics (see Tables A. 1-2).


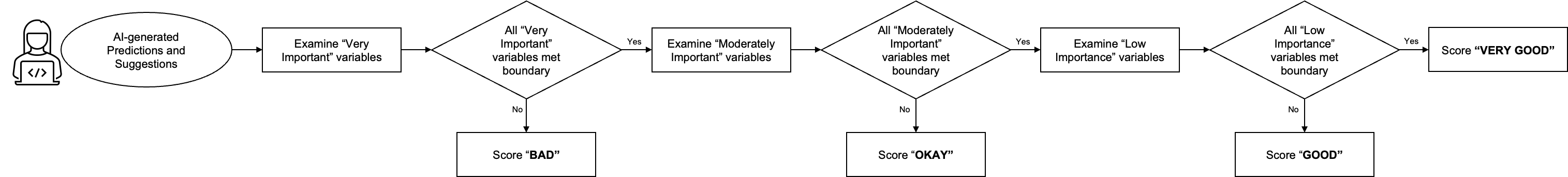


**Figure 1. 1.** “Expert-in-the-loop” Workflow

**Table 1. 1.** "Hard" boundaries for the ketogenic diet group.

| **Variables** | **Importance Rank** | **Boundary** |
| --- | --- | --- |
| Net Carb | Very important | 20-50 |
| Keto Ratio (calculated) | Very important | ≥ 1.5 |
| Weight | Very Important | ± 5 lbs. |
| Blood Glucose | Very important | 70-130 |
| Protein | Moderately Important | ≥ minimum protein |
| Fat | Moderately Important | ≥ minimum fat |
| Intake Calories (calculated) | Moderately Important | Lower than calorie goal |
| Blood Ketone | Moderately Important | ≥ 0.5 |
| Activity Calories | Low Importance | < 500 kcal + intake calories |
| Steps | Low Importance | ≥ 6000 |

**Table 1.2.** "Hard" boundaries for the low-fat diet group.

| Variables | Importance Rank | Boundary |
| --- | --- | --- |
| Carb | Moderately Important | < 65% calories from carb |
| Protein | Moderately Important | ≥ minimum protein |
| Fat | Very Important | < maximum fat |
| Intake Calories | Very Important | Lower than calorie goal |
| Activity Calories | Low Importance | < 500 kcal + intake calories |
| Steps | Low Importance | ≥ 6000 |
| Weight | Very Important | ± 5 lbs. |
| Blood Glucose | Very Important | 70-130 |

## **1.2. Translate A.I. Predictions and Suggestions to Deliverable Messages**

There were three components of daily text messages (1) a meal plan example, (2) tailored motivational messages, and (3) a daily step goal.

The meal plan example was created using the linear programming approach. We generated a list of five food groups - meat, fruits, vegetables, nuts and seeds, and saturated fats - with food items available from the ADA website. The objective of the linear programming approach was to ensure the variety of food items while meeting the daily nutrient needs and daily caloric intake limits for ketogenic diet and low-fat diet participants. Specifically, each individual had unique constraints considered while building the linear programming model. The linear programing algorithm selected the food items and serving sizes from different food groups to create a personalized meal plan (Figure A.2. shows a sample meal plan that is provided to the subjects of the intervention group).


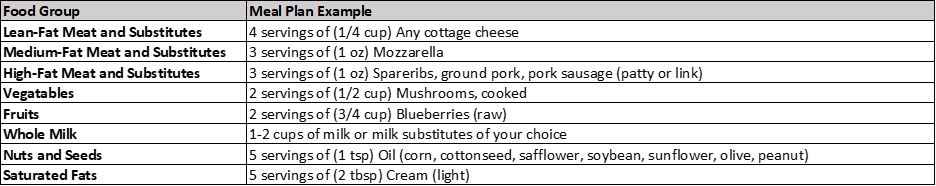


**Figure 1.2.** Daily Message: Meal Plan Example

Prior to the initiation of the intervention, the interventionist generated a motivational message pool consisting of 200+ messages that covered various domains including “Positive Feedback”, “Carbohydrate”, “Protein”, “Fat”, “Fiber”, “Overall Nutrition”, “Self-monitoring”, and “Exercise”. During the intervention, the interventionist evaluated areas need to be improved for each individual and then chose a message from the relevant domain to be sent.

To establish a daily step goal, we utilized the based on the 70^th^ percentile rank of the last 10 days step data and sent together with an exercise motivational quote.

**Table 1.3.** Example of AI suggestions for one ketogenic diet group participant

|  | **Net Carb** | **fat** | **protein** | **intake calories** | **activity calories** | **steps** | **glucose** | **ketone** | **weight** |
| --- | --- | --- | --- | --- | --- | --- | --- | --- | --- |
| Last observation | 39 | 45.2 | 104.1 | 1064 | 1009 | 5253 | 134 | 0.2 | 199.2 |
| AI Suggestion | 30 | 135 | 60 | 1064 | 1008 | 6000 |  |  |  |
| Predicted Outcome |  |  |  |  |  |  | 110 | 2.4 | 197.6 |
| Keto Ratio (Last Observation) | | 0.3 (vs. 1.5) | | | | | | | |
| Keto Ratio (AI Suggestion) | | 1.2 (vs. 1.5) | | | | | | | |
